# Response of root and root hair phenotypes of cotton seedlings under high temperature revealed with RhizoPot

**DOI:** 10.3389/fpls.2022.1007145

**Published:** 2022-11-08

**Authors:** Cong Fan, Mingyu Hou, Peng Si, Hongchun Sun, Ke Zhang, Zhiying Bai, Guiyan Wang, Cundong Li, Liantao Liu, Yongjiang Zhang

**Affiliations:** ^1^ College of Agronomy, State Key Laboratory of North China Crop Improvement and Regulation/Key Laboratory of Crop Growth Regulation of Hebei Province, Hebei Agricultural University, Baoding, China; ^2^ College of Life Science, Hebei Agricultural University, Baoding, China

**Keywords:** cotton, high temperature, *in situ* root, root dynamics, root hair, RhizoPot

## Abstract

Driven by the increase in its frequency and duration, high temperature weather is increasingly seriously affecting crop development. High temperature inhibits the leaf development, flowering, and pollination of cotton, but its effects on the roots and root hair phenotypes and lifespans remain unclear. Thus, this study selected the two cotton varieties Nongda 601 (ND) and Guoxin 9 (GX) as materials and adopted the RhizoPot, an *in situ* root observation system, to investigate the effects of high temperature (38°C day and 32°C night) on the growth dynamics of the aboveground parts and root phenotypes of cotton at the seedling stage. The results showed that high temperature reduced the net photosynthetic rate and chlorophyll content, decreased the dry matter accumulation and transfer to the root, and lowered the root-shoot ratio (R/S ratio). The root phenotypes changed significantly under high temperature. After 7 d of high temperature stress, the root lengths of ND and GX decreased by 78.14 mm and 59.64 mm, respectively. Their specific root lengths increased by 79.60% and 66.11%, respectively. Their specific root surface areas increased by 418.70 cm^2^·g^-1^ and 433.42 cm^2^·g^-1^, respectively. Their proportions of very fine roots increased to 99.26% and 97.16%, respectively. After the removal of high temperature (RHT), their root lengths tended to increase, and their proportions of very fine roots continued to increase. The root hairs of ND and GX were also significantly affected by high temperature. In particular, the root hair densities of ND and GX decreased by 52.53% and 56.25%, respectively. Their average root hair lengths decreased by 96.62% and 74.29%, respectively. Their root hair lifespans decreased by 7 d and 10 d, respectively. After the RHT, their average root hair lengths failed to recover. A principal component analysis indicated that the root architectures were significantly affected by root hair density, average root hair length, specific root length, and specific root surface area under high temperatures. In summary, cotton adapts to high temperature environments by increasing the specific root length, specific root surface area, and the proportions of very fine roots, and reducing the lifespan of root hairs.

## Introduction

Abiotic stresses, such as high temperature, drought, salt, chemical toxicity, and oxidative stress, pose serious threats to agricultural production (Wang et al., 2003). High temperature weather is increasing in both frequency and duration due to emissions of greenhouse gases, which subsequently affects crop development (Luo, 2011). Studies have pointed out that if global warming exceeds the pre-industrial level by 1.5°C, extreme high temperature weather will further intensify (Hoegh-Guldberg et al., 2019), thus, hindering crop growth and development, reducing crop yield and quality, and even resulting in complete crop failure (Fahad et al., 2017; Li et al., 2020; Parker et al., 2020). This is mainly because high temperature can easily cause the plant defense system to fail, making the plants more vulnerable to pathogens and pests and inhibiting the accumulation of photosynthetic products (Kim et al., 2022).

Cotton (*Gossypium hirsutum L.*), as a major cash and fiber crop, originated in tropical and subtropical regions and prefers warmth and light. Temperature has an extremely significant effect on cotton growth and development, and it can affect its growth and maturation and regulate its phenological development and rate of biomass accumulation (Xu et al., 2017). Temperature beyond a certain range (35°C) seriously affects the germination and elongation of pollen tubes (Zahid et al., 2016), causes indehiscent anthers and sterile pollen, and reduces the single boll weight, which ultimately leads to a decrease in crop yield (Zhang et al., 2022). Each time the daily maximum temperature rises by 1°C, the lint yield will decrease by 110 kg/hm^2^ (Gao et al., 2021). The current temperature is close to or above the optimal temperature for cotton growth and yield (30°C) (Majeed et al., 2021).

High temperature weather may alter the resource allocation of shoots and roots. Compared with the shoot parts, root structures and their responses to temperature changes through interactions are rarely explored (Luo et al., 2020). Roots are a major organ responsible for maintaining plants and absorbing nutrients (Lynch, 2011). Their morphological and physiological characteristics are closely related to stress resistance (Joshi et al., 2016). Root growth is a dynamic process that is facilitated by suitable temperatures (Mai et al., 2018; Gavelienė et al., 2022). When the optimal temperature is 22–30°C, a temperature of 32–40°C will inhibit root distribution and growth (Zahid et al., 2016). The optimal temperature range promotes an increase in the root-shoot ratio (R/S ratio) (Koevoets et al., 2016), but a temperature above the optimal temperature will reduce the absorption of water and nutrients by the roots and weaken their resistance to abiotic stresses (Luo et al., 2020). However, further research is still needed to clarify the development of cotton roots and the responses of their root hair phenotypes under high temperature.

The root phenotypes of crops can be altered to improve their high temperature resistance, which constitutes an important phenotypic characteristic. High temperature not only inhibits root development (Calleja-Cabrera et al., 2020; Hund et al., 2008) but also affects the absorption of water by the roots and accelerates root senescence, causing the lignified roots to elongate to almost the tip and resulting in a reduction in the root absorption area and rate of nutrient absorption (Zhen et al., 2020). Martins et al. (2017) showed that, as the temperature increases, the roots could elongate faster to protect the meristems. Previous research showed that the root length and number of cotton roots increased significantly at 35°C (McMichael and Quisenberry, 1993; McMichael and Burke, 1994), but when the temperature exceeded 35°C, both the main root and lateral root lengths were shortened (McMichael et al., 1996). After subjecting 64 different varieties of soybean to high temperature treatment (40°C day and 32°C night), Alsajri et al. (2019) found that the root length, root surface area, and root volume all decreased significantly. Under high temperature stress, the root diameter and root cortex thickness of rice were both significantly inhibited (Zhen et al., 2020). A study also revealed that high temperatures reduced the root dry weight and R/S ratio but increased the specific root length, specific root surface area, and specific root volume (Tahir et al., 2008). Fine roots (roots<2 mm in diameter) are the most active part of the whole root system (Comas et al., 2000; Eissenstat et al., 2000) and serve as the main pathway for the absorption of water and nutrients and the regulation of plant growth (Zeleznik and Dickmann, 2004; Zhang et al., 2020). Studies have shown that the growth rate of fine roots increases with increasing soil temperatures (Mahmud et al., 2019). Therefore, it is necessary to explore the dynamic changes in cotton roots under high temperature stress to manage extreme high temperature weather in the field and provide important references for production practice.

Root hairs are formed through the elongation of root epidermal cells. They increase the contact area between the roots and soil and improve the efficiency of water and nutrient absorption (Bates and Lynch, 2001; Bengough et al., 2011). The growth and development of root hairs are affected by abiotic stresses, such as high temperature and drought, and the insufficiency of nutrients, such as phosphorus (P) and nitrogen (N) (López-Bucio et al., 2003; Wei et al., 2016). For example, temperature-sensitive *Arabidopsis thaliana* seedlings were unaffected at normal temperature (20°C) but failed to form root hairs at elevated temperature (30°C), which manifests as a reduction in the root hair length and density (Kim et al., 2021). The root hair number of trifoliate orange decreased at 40°C–45°C (Mohammad and Shiraishi, 2000). N stress significantly shortened the root hair lifespan of cotton but greatly increased its root hair density and length (Zhu et al., 2022). Under low P stress, cotton responded to P deficiency by extending its root hair lifespan and increasing its specific root length and lateral root branch density (Zhang et al., 2021). Drought stress accelerates the death of fine roots and root hairs, and cotton has adapted to such external environments by developing more fine roots and longer root hairs (Xiao et al., 2020). However, it is still unclear how the root hairs of cotton respond to high temperature. In this context, exploring the root hairs and root hair lifespan of cotton under high temperature is of great value to clarify the physiological mechanisms that underlie the high temperature resistance of cotton.

In summary, existing studies on the effect of high temperature on cotton mainly focus on the phenotypes of aboveground parts but rarely touch upon the responses of roots, root hair phenotypes, or their lifespans under high temperature. It is highly necessary to conduct dynamic research on roots, since it facilitates dynamic observation of the characteristics of changes in root phenotypes. Traditional root research methods, such as the digging method (Cheng et al., 2009), the soil coring method (Gahoonia and Nielsen, 1991), and the soil block method (Oliveira et al., 2005), cannot realize dynamic observation because they all require destructive sampling to separate the roots from culture soil. In addition, traditional methods are time-consuming and labor-intensive and cause substantial damage to roots, making it difficult obtain complete roots. Alternatively, X-ray computed tomography (CT) and magnetic resonance imaging (MRI) support the dynamic observation of roots and are widely used to study root phenotypes (Kurogane et al., 2021; Li et al., 2022). However, both methods are extremely expensive and limited by container size and substrate type. Our laboratory independently developed the RhizoPot, an *in situ* root observation system, which is highly efficient, inexpensive, simple to operate, and has high imaging resolution. It has proven useful in studies on cotton under N (Zhu et al., 2022), drought (Xiao et al., 2020), and P (Zhang et al., 2021) stresses. A RhizoPot can obtain continuous lossless images of root phenotypes, and was employed in this study to investigate the dynamic response characteristics of the root phenotypes of two cotton varieties under high temperature and clarify the effects of high temperature on the dynamic changes in cotton roots and the morphology and lifespan of root hairs. The results increase our understanding of the characteristics of cotton root phenotypes and longevity responses under high temperature stress, and will facilitate the breeding of cotton varieties that are resistant to high temperature.

## Materials and methods

### Materials and system

**Figure 1:**
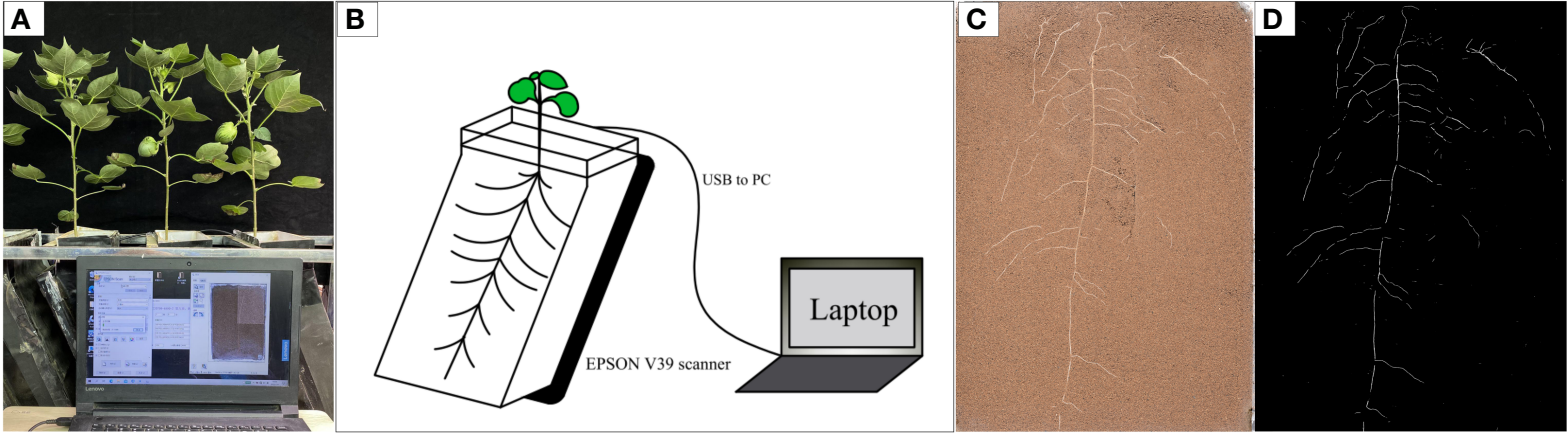
Schematic diagram of the *in situ* root observation system RhizoPot **(A)**, RhizoPot growth imaging device **(B)**, *In situ* root system image **(C)**, rendering of the segmented *in situ* root image **(D)**.

The experiment was conducted in the phytotron of Hebei Agricultural University (Baoding City, Hebei Province, China, 38.85° N, 115.30° E) from April to October 2021 (**Figure 1A**). Two local commercial cotton cultivars, Nongda 601 (ND) and Guoxin 9 (GX), were used in this study. The soil was sampled from the topsoil layer (0–20 cm) at the Experimental Station of Hebei Agricultural University (Baoding City, Hebei Province, China, 38.85° N, 115.30° E). The soil was pH 7.20; organic matter content, 16.57 mg·kg^-1^; total N, 1.23g·kg^-1^; alkali-hydrolysable N, 77.67 mg·kg^-1^; available phosphorus, 16.54 mg·kg^-1^; and available potassium, 129.32 mg·kg^-1^. After air drying, the soil was filtered through a 2 mm sieve to remove pebbles and large solid clods. The filtered soil was then mixed evenly by a weight ratio of soil: sand = 4:1 (v/v) to prepare a mixed substrate. The substrate was loaded into a RhizoPot, which was a growth vessel that we designed and assembled using transparent acrylic plates. A flatbed scanner (Epson Perfection Version 39, Suwa, Japan) used to collect images, and a laptop was used to control the operations of a scanner. The scanner was fixed to the inclined surface of the vessels. The outer wall of the RhizoPot was covered with a layer of black cardboard to prevent the exposure of roots to light (**Figure 1B**) (Xiao et al., 2020; Zhang et al., 2021). The mixed substrate weighed 6.5 kg in each RhizoPot.

### Growth conditions and treatments

Phytotron conditions: normal temperature CK, 26°C day and 20°C night; time: 14 h/10 h; light intensity: 600 μmol·m^-2^·s^-1^; relative water content of substrate: 45%–50% (Zhang et al., 2021). First, a wet towel was used to accelerate germination. Full seeds that germinated consistently were selected and sown in the RhizoPot. The seeds were 1 cm near the scanner side at a depth of 3 cm. High temperature (HT, 38°C day and 32°C night) treatment was started when the cotton grew to six leaves. The test treatments were as follows: CK + ND, normal temperature and Nongda 601 (CKND); CK + GX, normal temperature and Guoxin 9 (CKGX); HT + ND, high temperature and Nongda 601 (HTND); HT + GX, high temperature and Guoxin 9 (HTGX). After 7 d of high temperature treatment, the normal temperature (CK, 26°C day and 20°C night) was restored through the removal of high temperature (RHT). A total of 20 RhizoPot systems were employed. Each treatment was prepared with five replicates.

### Determination methods

#### Determination of the morphological indicators of aboveground parts

The morphological indicators of aboveground parts were determined at 0 d, 1 d, 3 d, 5 d, and 7 d after high temperature treatment and 4 d and 7 d after the RHT. The height from the cotyledon node to the stem tip was measured as the plant height using a ruler. The stem diameter 1 cm above the cotyledon node was measured with a Vernier caliper. The leaf area was measured by the length and width coefficient method.

#### Determination of the physiological indicators of aboveground parts

The net photosynthetic rate of the top third leaf on the stem was determined at 0 d, 1 d, 3 d, 5 d, and 7 d after high temperature treatment and 4 d and 7 d after RHT using a portable photosynthetic system (LI-6400XT; LI-COR, Lincoln, NE, USA). The light intensity was set at 600 μmol·m^-2^·s^-1^. The maximum photochemical efficiency (Fv/Fm) and the actual photochemical quantum yield (ΦPSII) were measured using a portable modulated chlorophyll fluorimeter (PAM-2500, Walz, Germany) at the same location where the photosynthetic parameters were determined. The relative chlorophyll content (SPAD) was measured using a chlorophyll meter (SPAD-502, Konica-Minolta, Tokyo, Japan). The leaf measured was the same as that measured for photosynthesis.

### *In situ* root observation

*In situ* root collection was performed each day starting with high temperature treatment. The resolution of images scanned was 1,200 and 4,800 dpi (**Figure 1C**). The whole observation window was scanned for 1,200 dpi images. The observation window was evenly divided into four parts for separate scanning for 4,800 dpi images (Xiao et al., 2020; Zhu et al., 2022). Scanned 1,200 dpi *in situ* root images were used for image segmentation using the improved DeepLab v3 + (Shen et al., 2020). The extracted roots were white, and the substrate was black (**Figure 1D**). The segmented root images were analyzed using WinRHIZO software (Reg 2009, Instruments Region, Inc., Québec City, Canada). The root length (RL, cm), average root diameter (AD, mm), root surface area (RSA), and root volume (RV, cm^3^) were obtained by analyzing the root images scanned. The roots were divided into fine roots (less than 2 mm in diameter) and very fine roots (less than 0.5 mm in diameter) based on the average root diameter obtained. The root length density (RLD, cm · cm^-3^) was calculated from the following formula (Zhang et al., 2021):


RLD=RL/A×DOF

where A denotes the area of the observation window (cm^2^), and DOF denotes the soil thickness observable by the RhizoPot, which was set at 0.25 cm in this study.

The *in situ* root images with a resolution of 4,800 dpi were analyzed for root hair phenotype traits using Adobe Photoshop 2020 (Adobe, San Jose, CA, USA). Three points were randomly selected on each image. The average root hair length (ARHL, mm) was measured using the scale of Adobe Photoshop 2020. The number of root hairs within 1 mm^2^ of each point was measured to obtain the root hair density (RHD). The root hairs were considered senescent and dead when they gradually turned from white to yellow and became twisted (Xiao et al., 2020; Zhang et al., 2021).

### Sample collection and parameter determination

The cotton plants were sampled after 7 d of high temperature treatment and 7 d of recovery. First, a plant was divided by the cotyledon node into its aboveground parts and belowground parts, and the fresh weights were measured. The dry weights were measured after the samples were dried to a constant weigh in an 85°C oven. A root flushing platform designed by our laboratory was used to rinse the substrate in the culture pan with a tap under a certain pressure to obtain clean whole roots that were free of impurity. The roots were then scanned using an Epson 10000 XL scanner with a resolution of 600 dpi. WinRHIZO software (Reg 2009, Instruments Region, Inc., Québec City, Canada) was used for analysis to obtain the root length, average root diameter, root volume, and root surface area. The specific root length, specific root surface area, and specific root volume were calculated:


$$\text{Specific root length}(\text{cm}\cdot {\text{g}}^{-1})=\text{root length}/\text{root dry weight}$$

$$\text{Specific root surface area}({\text{cm}}^{2}\cdot {\text{g}}^{-1})=\text{root surface area}/\text{root dry weight}$$

$$\text{Specific root volume}({\text{cm}}^{3}\cdot {\text{g}}^{-1})=\text{root volume}/\text{root dry weight}$$

### Statistical analysis

Microsoft Excel 2010 (Redland, WA, USA) was used for data statistics, sorting, and analysis. SPSS 21.0 (IBM, Inc., Armonk, NY, USA) was adopted for a one-way analysis of variance (ANOVA) and a correlation analysis between the treatment groups. The Kaplan-Meier method was employed for survival analysis (Kaplan and Meier, 1958). The average root hair lifespan was equal to the average survival time, and the median root hair lifespan (i.e., time to 50% survival) was estimated and used to plot survival curves (Xiao et al., 2020). A correlation analysis and principal component analysis (PCA) were performed to clarify the relationships between these traits. GraphPad Prism 8.0 (San Diego, CA, USA) and Origin Pro2022b (OriginLab, Northampton, MA, USA) were used for drawing.

## Results

### Effects of high temperature and 7 d recovery on the morphology and relative chlorophyll content of the aboveground parts

**Figure 2:**
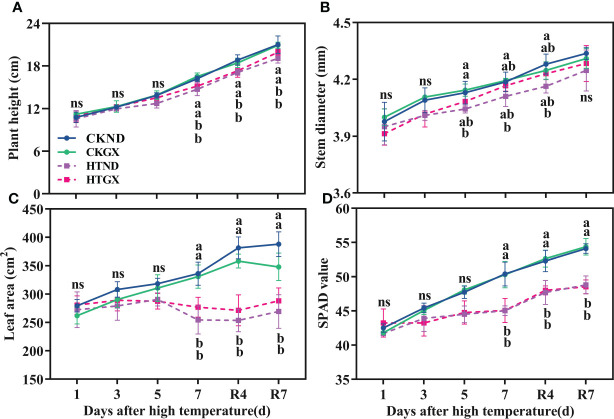
Changes in plant height **(A)**, stem diameter **(B)**, leaf area **(C)**, and relative chlorophyll content **(D)** of two cotton varieties under normal conditions and high temperature and after 7 d of recovery. The means of three replicates ± standard error are depicted. For each trait, bars with the same letter are not significantly different according to Duncan’s test at a *p*<0.05 threshold. ns, not significant (*p*>0.05).

High temperature significantly inhibited the development of the aboveground parts of cotton (**Figure 2**). The plant height, stem diameter, leaf area, and SPAD all tended increase as the duration of high temperature was extended, and the gap gradually widened at 5 d of the high temperature treatment. At 5 d, compared with those of the control, the plant heights of ND and GX under high temperature treatment decreased by 8.17% and 2.64%, respectively (**Figure 2A**); their stem diameters decreased by 2.10% and 1.26%, respectively (**Figure 2B**). High temperature aggravated cotton senescence, resulting in leaf abscission. The leaf areas of HTND and HTGX decreased by 8.77% and 7.46%, respectively (**Figure 2C**), and their SPAD values decreased by 6.77% and 6.86%, respectively (**Figure 2D**). At 7 d, compared with those of the control, the plant heights of HTND and HTGX decreased significantly by 9.05% and 8.08%, respectively; their leaf areas decreased by 24.29% and 16.33%, respectively, and their SPAD values decreased by 10.65% and 10.40%, respectively. The difference was significant in each case (*p*<0.05). In contrast, their stem diameters decreased by 1.81% and 0.56%, respectively. All the indicators increased after the RHT.

### Effects of high temperature and 7 d recovery on the net photosynthetic rate and chlorophyll fluorescence parameters

**Figure 3:**
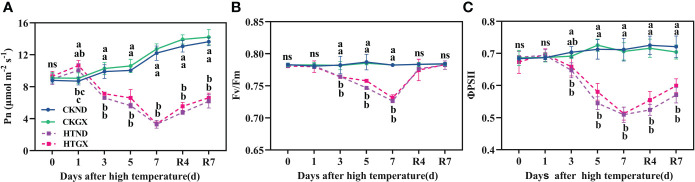
Changes in net photosynthetic rate (Pn) **(A)**, maximum photochemical efficiency (Fv/Fm) **(B)**, and actual photochemical quantum yield (ΦPSII) **(C)** of two cotton varieties under normal conditions and high temperature and after 7 d of recovery. The means of three replicates ± standard error are depicted. For each trait, bars with the same letter are not significantly different according to Duncan’s test at a *p*<0.05 threshold. ns, not significant (*p*>0.05).

With the extension of duration of high temperatures, the net rate of photosynthesis (Pn) of HTND and HTGX both tended to decrease (**Figure 3A**). At 1 d, due to the short duration of high temperature, the net rate of photosynthesis of HTND and HTGX, compared with those of the control, increased significantly by 48.56% and 17.55%, respectively (*p*<0.05). At 2 d-7 d, the net rate of photosynthesis of HTND and HTGX abruptly decreased. Compared with those of the control, the net rate of photosynthesis of HTND and HTGX decreased significantly by 19.38% and 30.89% (*p*<0.05) at 3 d, respectively, and by 69.78% and 73.77% (*p*<0.05) at 7 d, respectively. Within 7 d after the RHT, the net rate of photosynthesis tended to increase, but it was still lower than that of the control.

PSII maximum photochemical efficiency (Fv/Fm) reflects the maximum photosynthetic potential of plants and is an important criterion on the occurrence of photoinhibition during photosynthesis. High temperature treatment significantly reduced the Fv/Fm of ND and GX (*p*<0.05) (**Figure 3B**). Compared with that of the control, the Fv/Fm of ND decreased significantly by 2.30%, 5.12%, and 7.16% at 3 d, 5 d, and 7 d of the high temperature treatment, respectively (*p*<0.05), while that of GX decreased significantly by 2.26%, 3.52%, and 6.55%, respectively, (*p*<0.05). Fv/Fm rapidly increased after the RHT. There was no significant difference between any treatment and the control at 7 d after RHT.

PSII actual photochemical quantum yield (ΦPSII) reflects the actual photochemical efficiency of a photoreaction. High temperature treatment reduced ΦPSII (**Figure 3C**), which was significantly lower than that of the control at each treatment period (*p*<0.05). Compared with those of the control, the ΦPSII of HTND at 3 d, 5 d, and 7 d decreased significantly by 7.97%, 23.56%, and 28.48% (*p*<0.05), respectively. Those of HTGX decreased significantly by 4.69%, 20.0%, and 27.45% (*p*<0.05), respectively. The changing trend of ΦPSII after the RHT was consistent with that of Fv/Fm. At 7 d after the RHT, the ΦPSII of HTND and HTGX were still significantly lower than those of the control and decreased by 20.90% and 14.93%, respectively (*p*<0.05).

### Effects of high temperature and 7 d recovery on the biomass and R/S ratio

High temperature significantly inhibited the growth of cotton plants. After high temperature treatment, the fresh and dry weights of the aboveground and belowground parts decreased significantly (*p*<0.05) (**Table 1**). Compared with those of the control, the fresh and dry weights of the aboveground parts of HTND decreased by 24.10% and 22.90%, respectively. However, after 7 d recovery, there was no significant difference between them. In contrast, those of HTGX decreased by 26.08% and 28.60%, respectively. After the high temperature treatment, the fresh and dry weights of the belowground parts of ND decreased by 27.88% and 45.23%, respectively, while those of GX decreased by 26.39% and 38.04%, respectively. High temperature treatment caused the R/S ratios of the two varieties to significantly decrease by 29.56% and 15.89%, respectively (*p*<0.05).

**Table 1:** Plant biomass and R/S ratio under normal and high temperatures.

Parameters	ND		GX	
	CK	HT	CK	HT
Shoot fresh weight(g)	13.51 ± 1.74ab	10.25 ± 1.92c	15.39 ± 0.86a	11.37 ± 1.65bc
Root fresh weight(g)	9.82 ± 2.47ab	9.08 ± 0.68b	9.87 ± 1.22a	9.26 ± 0.69ab
Total fresh weight(g)	23.33 ± 3.78ab	19.34 ± 2.42b	25.26 ± 2.05a	20.64 ± 2.16ab
Shoot dry weight(g)	4.02 ± 0.21ab	3.10 ± 0.89b	1.50 ± 0.72a	3.31 ± 0.64ab
Root dry weight(g)	2.13 ± 0.09a	1.17 ± 0.38b	2.24 ± 0.29a	1.40 ± 0.54b
Total dry weight(g)	6.15 ± 0.21ab	4.27 ± 1.26b	6.74 ± 1.00a	4.60 ± 1.71b
R/S	0.53 ± 0.04a	0.37 ± 0.09c	0.50 ± 0.02ab	0.42 ± 0.08bc

Depicted are the means of three replicates ± standard error. Different letters indicate that there are significant differences between different treatments (*p* < 0.05).

### Effects of high temperature and 7 d recovery on the roots (destructibility)

High temperature significantly inhibited the growth of cotton roots and reduced the root length, root surface area, root volume, and average root diameter (*p*<0.05) (**Table 2**). After high temperature treatment and the RHT, the root length of HTND decreased by 7.79% relative to that of the control, and its root surface area, root volume, and average root diameter decreased by 10.10%, 11.21%, and 4.50%, respectively. In contrast, those of HTGX decreased by 8.53%, 10.10%, 17.0%, and 6.54%, respectively.

**Table 2:** Cotton root parameters of two varieties under normal and high temperatures.

Parameters	ND		GX	
	CK	HT	CK	HT
Root length(cm)	7261.34a	6695.93a	7467.85a	6830.63a
Root surface area(cm^2^)	1389.08ab	1248.85c	1464.05a	1316.34bc
Root volume(cm^3^)	21.33ab	18.94b	23.06a	19.14b
Specific root length(cm·g^-1^)	3417.09b	6127.33a	3332.26a	5527.11b
Specific root surface area(cm^2^·g^-1^)	653.31b	1157.16a	659.56b	1085.75a
Specific root volume(cm^3^·g^-1^)	10.03b	17.85a	10.49b	15.48a
Average root diameter(mm)	0.63a	0.60a	0.66a	0.61a

Depicted are the means of three replicates. Different letters indicate that there are significant differences between different treatments (*p* < 0.05).

Under high temperature treatment, the two varieties differed significantly from the control in terms of specific root length, specific root surface area, and specific root volume (*p*<0.05) (**Table 2**). High temperature treatment significantly increased the specific root length, specific root surface area, and specific root volume (*p*<0.05). Compared with those of the control, the specific root length, specific root surface area, and specific root volume of ND under high temperature treatment increased by 79.60%, 64.15%, and 78.10%, respectively. In contrast, those of GX increased by 66.11%, 433.42 cm^2^·g^-1^, and 5.20 cm^3^·g^-1^, respectively.

### Effects of high temperature and 7 d recovery on the dynamics of root development

**Figure 4:**
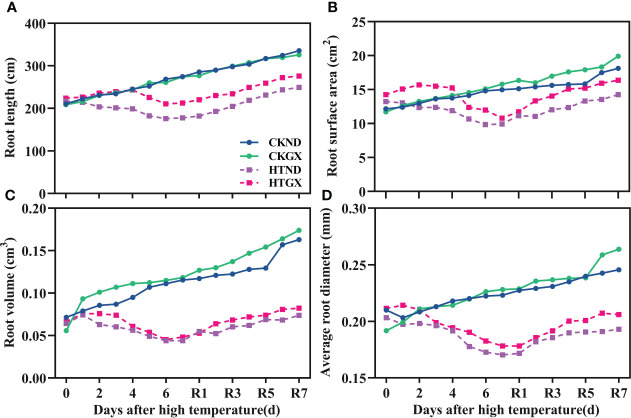
Changes in the root length **(A)**, root surface area **(B)**, root volume **(C)**, and average root diameter **(D)** of two cotton varieties under normal conditions and high temperature and after 7 d recovery.

After high temperature treatment, the root length, root surface area, root volume, and average root diameter of cotton all decreased significantly (*p*<0.05) (**Figure 4**). The root length of HTND decreased significantly by 27.74% and 35.31% at 5 d and 7 d, respectively, while that of HTGX decreased by 13.21% and 22.71%, respectively. The root surface areas of HTND and HTGX decreased by 24.59% and 15.13% at 5 d, respectively, and by 33.68% and 31.68% at 7 d, respectively. The root volumes of HTND and HTGX decreased by 54.21% and 52.23% at 5 d, respectively, and by 61.90% and 51.95% at 7 d, respectively. The average root diameters of HTND and HTGX decreased by 18.25% and 13.40% at 5 d, respectively, and by 23.64% and 21.91% at 7 d, respectively. After the RHT, the root length, root surface area, root volume, and average root diameter all tended to increase.

### Effects of high temperature and 7 d recovery on the root growth rate and root length density

**Figure 5:**
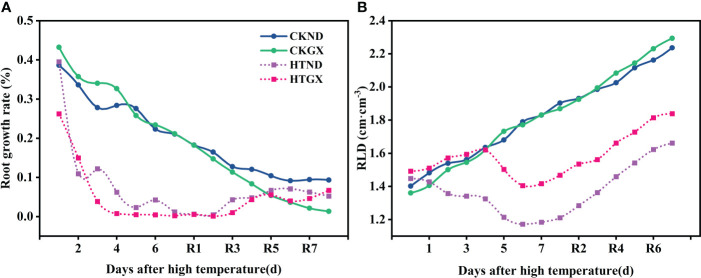
Changes in the root growth rate **(A)** and root length density **(B)** of two cotton varieties under normal conditions and high temperature and after 7 d recovery.

The changes in rates of root growth of two cotton varieties during treatment and 7 d after RHT were observed (**Figure 5A**). At 1d and 3 d, there was no change in the rate of root growth of HTND or HTGX relative to the control, and positive growth was observed in each case. The root growth rates of HTND and HTGX decreased by -0.018 and -0.004 at 5 d, respectively, and by -0.042 and -0.177 at 7 d, respectively. After the RHT, the root growth rates of HTND and HTGX tended to increase. The root growth rates of HTND and HTGX were 0.04 and 0.324 at 4 d after RHT, respectively, and 0.05 and 0.087 at 7 d, respectively. After high temperature treatment, the root length densities of ND and GX increased at 1 d, 3 d, and 5 d, similar to the trend presented by the root length density of the control. They began to decrease at 6 d of the high temperature treatment but increased again after the RHT (**Figure 5B**).

### Effects of high temperature and 7 d recovery on the proportions of fine roots and very fine roots

**Figure 6:**
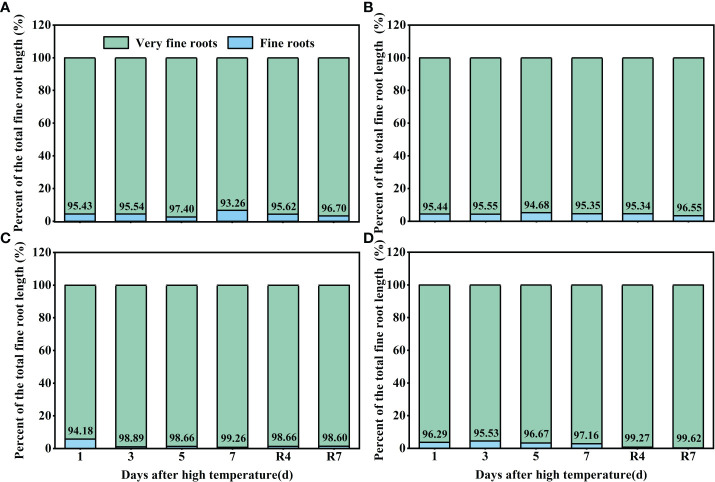
Changes in the proportions of fine roots and very fine roots of two cotton varieties under normal conditions and high temperature and after 7 d recovery (**A**: CKND; **B**: CKGX; **C**: HTND; **D**: HTGX).

Observation of the cotton roots indicated that most of the roots< 0.5 mm in diameter (very fine roots). High temperature increased the proportion of very fine roots. Under control conditions, the proportion of very fine roots of CKND was 93%–97%, and that of CKGX was 94%–96% (**Figures 6A, B**). At 1 d of the high temperature treatment, there was little difference between HTND and the control in the proportion of very fine roots. At 3 d, the proportion of very fine roots in the total root length was 98.89%. At 7 d, the proportion of very fine roots peaked (99.26%). After the RHT, the proportion of very fine roots did not decrease but remained around 98% (**Figure 6C**). In contrast, the proportion of very fine roots of HTGX was basically consistent with that of the control at 1 d-3 d, increased to 99.67% at 5 d, and decreased slightly to 97.16% at 7 d. However, after the RHT, it remained around 99% (**Figure 6D**). This indicated that, under high temperature treatment, very fine roots emerged in large quantities, or there was no increase in root diameter.

### Effects of high temperature on the root hair length, density, and survival

**Figure 7:**
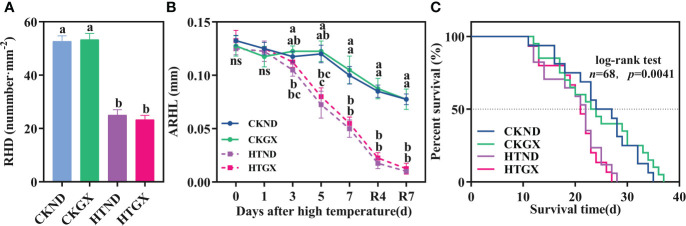
Changes in the root hair density **(A)**, average root hair length **(B)**, and root hair survival **(C)** of two cotton varieties under normal conditions and high temperature and after 7 d recovery. **(A)** values are the means of three replicates ± standard error, **(B)** values are the means of four replicates ± standard error, n, the number of root hairs used to draw the survival curve; The *p* -values indicate the statistical significance of the effect of high temperature stress on the root hair lifespan of cotton. For each trait, bars with the same letter are not significantly different according to Duncan’s test at a *p*<0.05 threshold. ns, not significant.

High temperature significantly inhibited the root hair density and root hair length of cotton and lowered the survival of its root hairs. After high temperature treatment, the root hair densities of ND and GX decreased significantly by 52.53% and 56.25% (*p*<0.05) relative to those of the control, respectively (**Figure 7A**). At 1 d of the high temperature treatment, neither ND nor GX differed significantly from the control in root hair length. The root hair length of HTND decreased significantly by 84.87%, 93.39%, and 96.62% relative to that of the control at 3 d, 5 d, and 7 d, respectively (*p*<0.05), while that of GX decreased significantly by 34.69%, 47.62%, and 74.29% (*p*<0.05), respectively (**Figure 7B**). Within 7 d after the RHT, the significant trend to decrease persisted (*p*<0.05).

**Figure 8:**
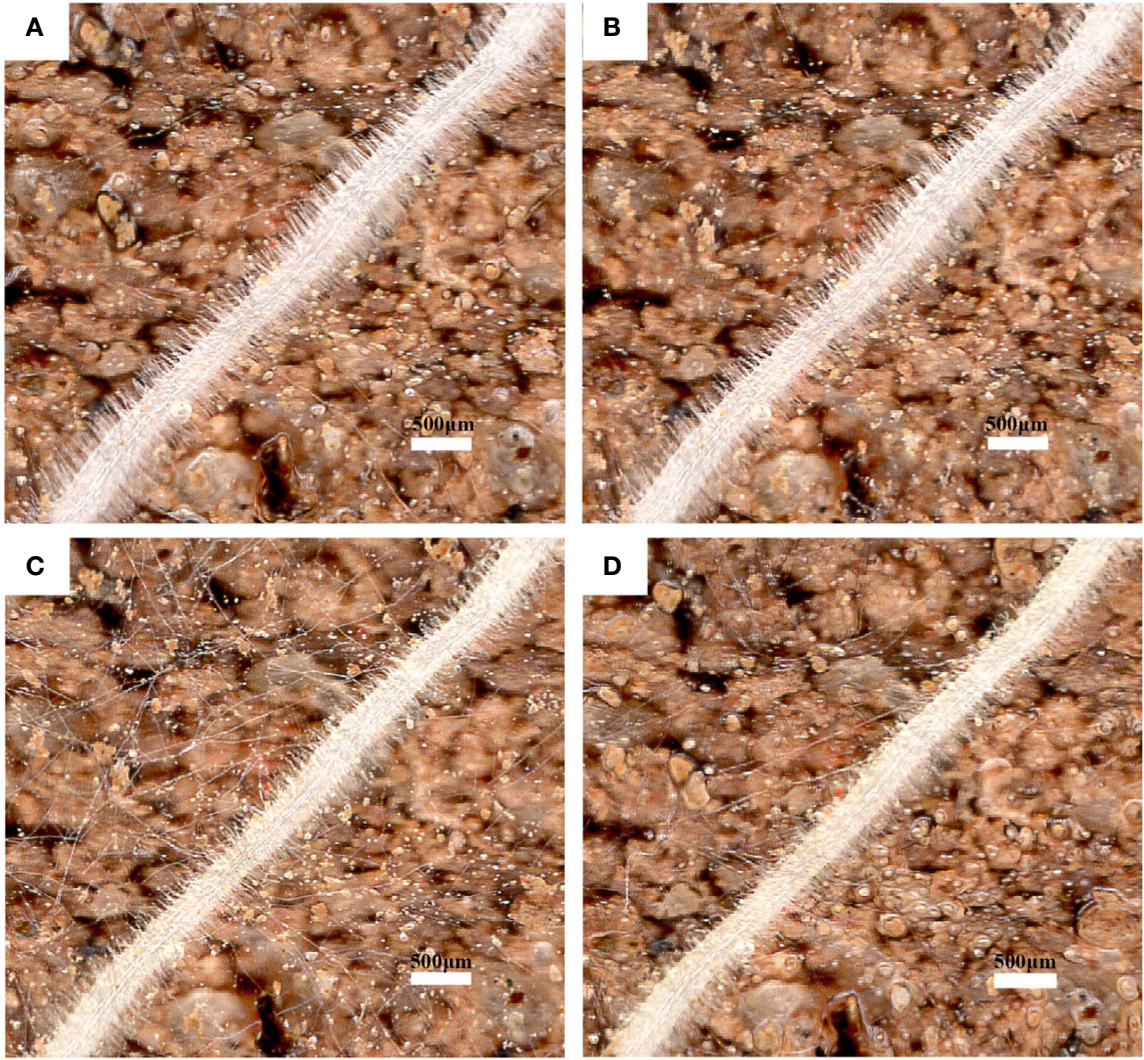
Images of the same root region of cotton root hairs under high temperature stress. Scale bar, 500 μm. Images shown are taken on 1d **(A)**, 3d **(B)**, 5d **(C)**, 7d **(D)**.

The root hair lifespan refers to the time from the emergence of root hairs to the occurrence of exterior twisting (**Figure 8**). The median lifespans of CKND and HTND were 27 d and 21.5 d, respectively, while those of CKGX and HTGX were 23 d and 21 d, respectively. High temperature aggravated the senescence of root hairs and caused the root hair lifespans of ND and GX to decrease significantly by 7 d and 10 d relative to those of the control, respectively (*p*<0.05) (**Figure 7C**).

### PCA and correlation analysis of cotton traits

**Figure 9:**
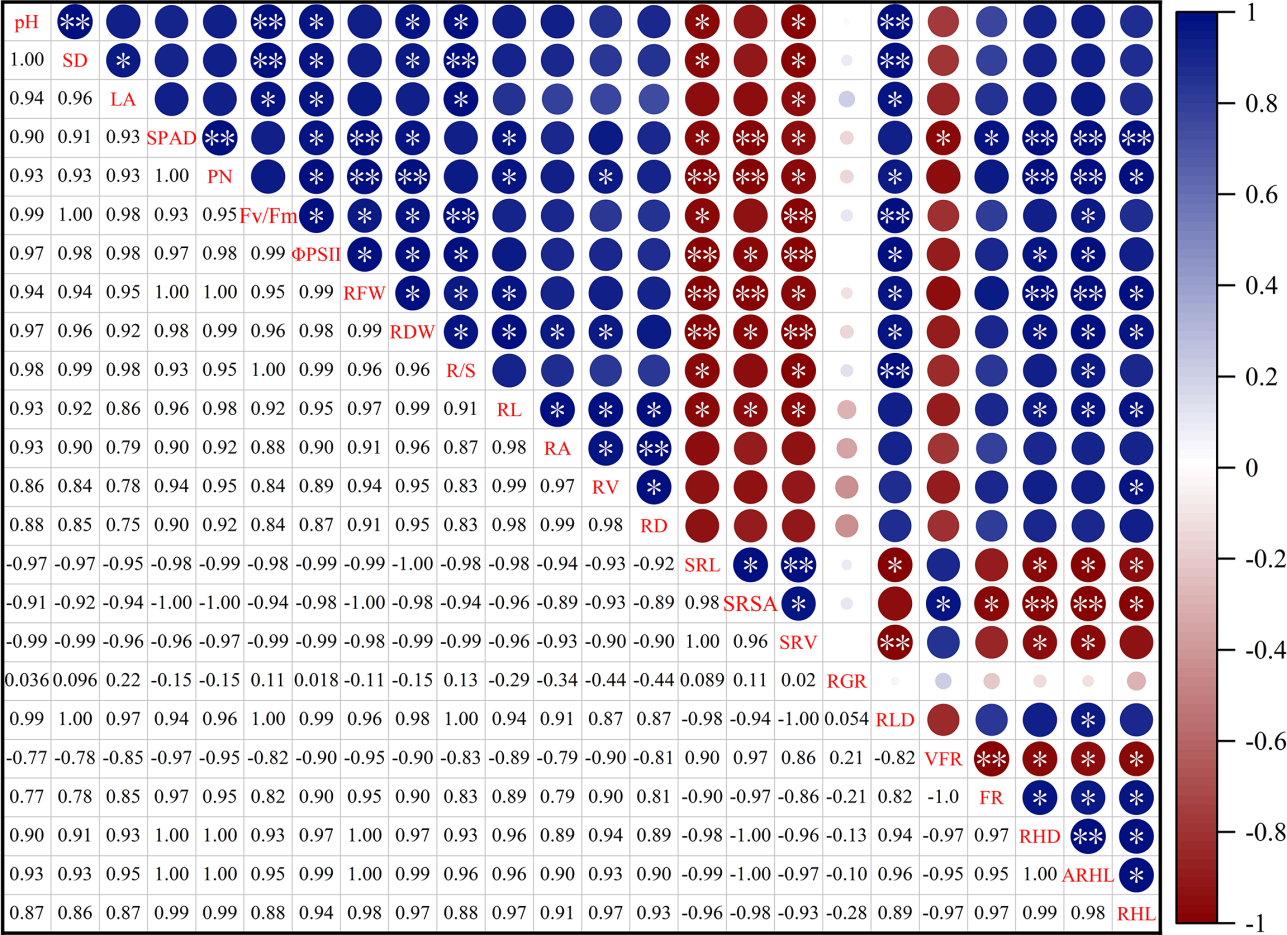
Pearson correlation matrix between the cotton traits. The level of significance of the correlations is indicated as follows: *p< 0.05; **p< 0.01. PH, plant height; SD, stem diameter; LA, leaf area; SPAD, spad value; Pn, net photosynthetic rate; Fv/Fm, maximum photochemical efficiency; ΦPsII, actual photochemical quantum yield; RFW, root fresh weight; RDW, root dry weight; R/S, root-shoot ratio; RL, root length; RA, root surface area; RV, root volume; RD, average root diameter; SRL, specific root length; SRSA, specific root surface area; SRV, specific root volume; RGR, root growth rate; RLD, root length density; VFR, proportion of very fine roots; FR, proportion of fine roots; RHD, root hair density; ARHL, average root hair length; RHL, root hair lifespan.

**Figure 10:**
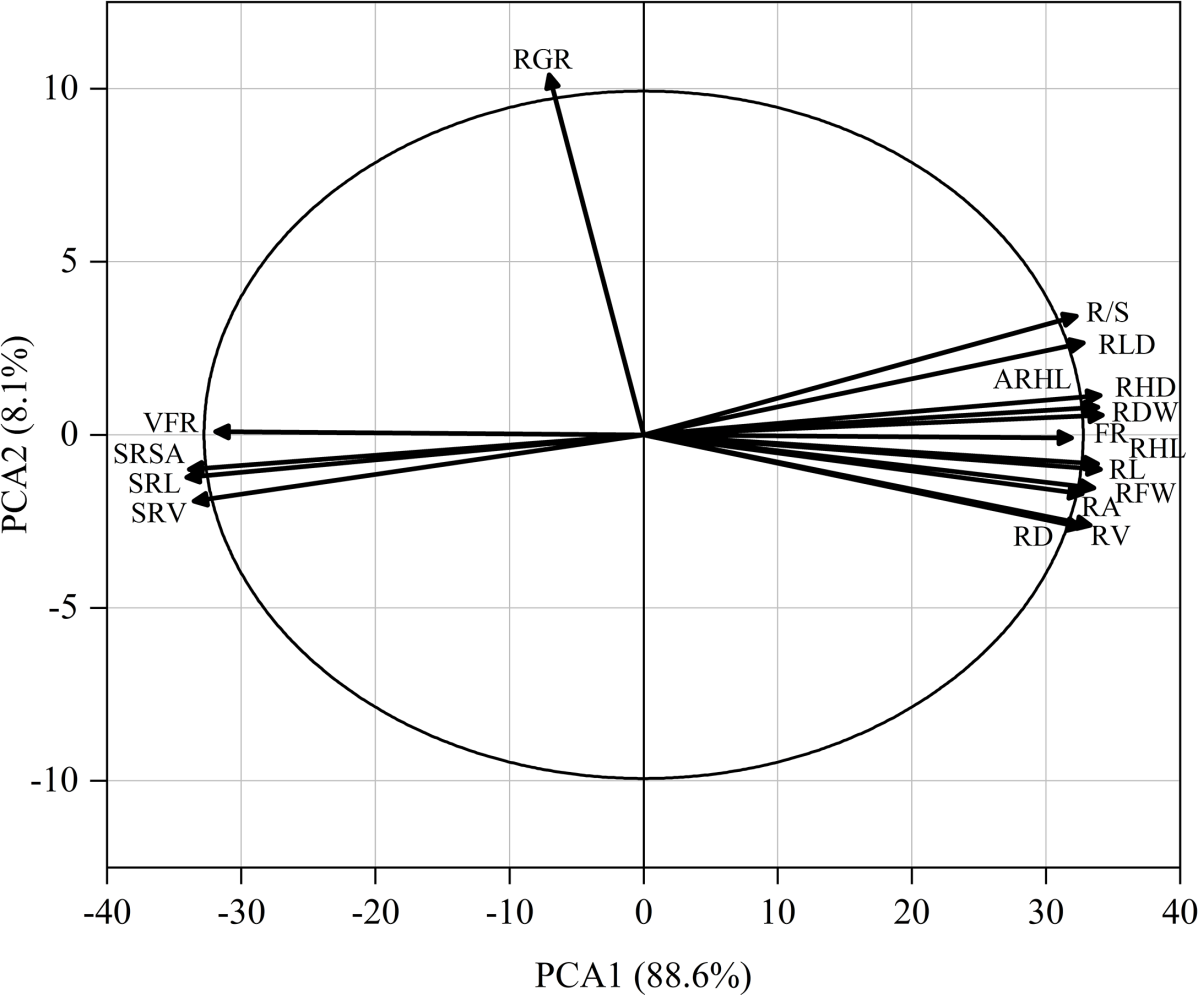
Principal component analysis of 17 root system indicators (abbreviated as in **Figure 9**).

As shown in **Figure 9**, the root hair lifespan significantly positively correlated with the average root hair length, root hair density, proportion of fine roots, root volume, root length, root dry weight, root fresh weight, net photosynthetic rate, and relative chlorophyll content but significantly negatively correlated with the specific root length and specific root surface area. The specific root length significantly positively correlated with the specific root surface area and specific root volume but significantly negatively correlated with the plant height, stem diameter, SPAD, net photosynthetic rate, Fv/Fm, ΦPSII, root fresh weight, root dry weight, R/S ratio, root length, root hair density, average root hair length, and root hair lifespan. The proportion of very fine roots significantly positively correlated with the proportion of fine roots. A PCA was performed on 17 root system indicators in this study (**Figure 10**). The rate of contribution of the first two principal components was 96.7%. Principal component 1 contributed 88.6%. In particular, the root length, proportion of fine roots, root hair density, average root hair length, root hair lifespan, and root dry weight were close to the positive direction of the x-axis, while the proportion of very fine roots, specific root length, and specific root surface area were in the negative direction of the x-axis. Principal component 2 contributed a smaller amount, accounting for 8.1% of total variation. The root growth rate substantially contributed to principal component 2.

## Discussion

### Effects of high temperature on the morphology and physiology of the aboveground parts

Abiotic stresses increasingly threaten existing ecological and agricultural systems across the globe. In reality, most biological temperature responses increase exponentially with temperature until they reach a thermal optimum. Plant roots perceive these stresses in the soil and adapt their architecture accordingly (Karlova et al., 2021; Moore et al., 2021). Plant growth is a process of continuous increase in weight and volume. This process is irreversible, but when exposed to high temperature, it undergoes a series of physiological and biochemical reactions, which ultimately lead to slow growth and even the arrest of growth until death. Previous research revealed that the plant height of cotton increased linearly with increasing temperature (Reddy et al., 2017) and that the leaf area index and the dry matter accumulation of the aboveground parts peaked at 35°C but began to decrease after 35°C (Virk et al., 2021). This study found that a high temperature of 38°C significantly reduced the plant height, stem diameter, leaf area, and SPAD of ND and GX (**Figure 2**). At the early stage of high temperature, the leaf growth decreased, and at 5 d, some leaves began to fall off, possibly because the high temperature accelerated leaf senescence, resulting in leaf abscission. This study also indicated that the dry and fresh weights of the aboveground parts decreased significantly after high temperature stress (**Table 1**), similar to the response of potato to high temperature (Taranet et al., 2018). The effect of high temperature on the dry matter accumulation was related to its effect on the photosynthetic rate. The photosynthetic rate of cotton decreased sharply at 35°C (Zahid et al., 2016; Moore et al., 2021). This study also discovered that high temperature reduced the photosynthetic rate (**Figure 3A**). This changing trend highly correlated with that of Fv/Fm, i.e., with the extension of high temperature duration, Fv/Fm and ΦPSII began to gradually decrease until the last day of stress (**Figures 3B, C**). This is consistent with the responses of other crops to high temperature stress. For example, the Fv/Fm and ΦPSII of melon (Weng et al., 2022) and soybean (Jumrani et al., 2017) are seriously inhibited and rapidly decreased under high temperature stress. After the RHT, the net photosynthetic rate and Fv/Fm both tended to increase. After 7 d of recovery, the Fv/Fm recovered to a normal level, and no irreversible damage was caused to the aboveground parts of cotton. Therefore, in this study, the response of aboveground parts to high temperature (HT) treatment (38°C day and 32°C night) showed obvious phenotypic and physiological characteristics of high temperature stress.

### Effect of high temperature on root development

An increase in temperature is beneficial to the root growth of plants within a suitable range, but it affects root development once beyond this range (Fonseca de Lima et al., 2021; Snider et al., 2022). A major function of roots is to absorb water and nutrients. However, high temperature stress weakens this function, thus, reducing the transportation of water and nutrients to the aboveground parts and further inhibiting plant growth and changing root architecture (Giri et al., 2017; Calleja-Cabrera et al., 2020; Tiwari et al., 2022). Studies on wheat have pointed out that high temperature stress reduces the root dry weight, root length, and R/S ratio; increases the specific root length, specific root surface area, and specific root volume; and decreases the number of roots (Benlloch-Gonzalez et al., 2014). A study on high temperature stress in cotton suggests that high temperature reduces the root surface area, root length, root volume, and average root diameter (Reddy et al., 2017). This study showed that the specific root length significantly positively correlated with the specific root surface area and specific root volume (**Figure 9**), after high temperature stress, the specific root length, specific root surface area, and specific root volume of cotton increased significantly, which was consistent with the results of studies on wheat (Tahir et al., 2008; Benlloch-Gonzalez et al., 2014). In contrast, the root length, root surface area, and root volume decreased significantly, suggesting that high temperature inhibited root growth (**Table 2**).

In this study, the root length was significantly and positively correlated with root surface area and root volume (**Figure 9**). This was possibly because high temperature inhibited the synthesis of endogenous hormones, such as brassinolide and auxin, in roots. At elevated temperatures, the root system also elongates to protect the meristem (Martins et al., 2017), while increasing the number of roots and decreasing the length of lateral roots (Alsajri et al., 2019). Root branching strength and average root diameter were highly sensitive to high temperature, which significantly increased the root branching strength, and the average root diameter decreased due to the increase in branching strength (Nagel et al., 2009). This could possibly occur because high temperature accelerates the division of root meristems and accelerates the development of lateral root primordia (Otsuka and Sugiyama, 2012). When the average root diameter decreases, it may change the acquisition of root nutrients and affect root development (Luo et al., 2020), thus, increasing root branching and distribution to adapt to abiotic stresses, such as high temperature (Zahid et al., 2016). Studies on grape lupine and sorghum found that high temperature inhibits root growth, changes the R/S ratio, and affects the root architecture (Pardales et al., 1992; Ribeiro et al., 2014; Mahmud et al., 2019; Gavelienė et al., 2022). This study clearly found that high temperature treatment significantly reduced the growth rate of roots by relying on the *in situ* root observation system RhizoPot (**Figure 5A**) and that the root growth was arrested at 4 d of the high temperature treatment but recovered at 2 d after the RHT.

### Reduction in root hair density, length, and lifespan by high temperature

A root hair is a top-closed tubular structure with epidermal cells that protrude outwards. Root hairs are closely fitted with soil, which greatly increases the root surface area and improves the efficiency of water and nutrient absorption (Peterson and Farquhar, 1996; Raven and Edwards, 2001). Root hairs consume little energy when absorbing nutrients from soil (Nestler and Wissuwa, 2016; Wang et al., 2021), and the growth and development of root hairs play a vital role in alleviating abiotic stresses, such as high temperature and drought (López-Bucio et al., 2003). Root hairs have a short lifespan of about 10 to 20 d, and their senescence is manifested as a death state of twisting and rotation (Hendrick and Pregitzer, 1992; Xiao et al., 2020; Zhu et al., 2022). The RhizoPot platform introduced in this study clearly displayed the whole process of root hairs from emergence to the state of twisting and rotation, which offers support for studies on the root hair phenotypes and lifespan of cotton under high temperature stress. This study found that root hair longevity is significantly and positively correlated with the mean root hair length and root hair density (**Figure 9**) after high temperature stress significantly reduced the root hair density of cotton (**Figure 7A**) and shortened its average root hair length (**Figure 7B**). The lifespan of root hairs was significantly reduced, and most of the root hairs twisted and died at the end of 7 d continuous high temperature treatment (**Figure 7C**). This result indicated that root hairs are highly sensitive to high temperature stress. However, this result was different from previous research that showed that root hair density and length both tend to increase under drought stress and low N and P stress (Xiao et al., 2020; Zhang et al., 2021; Zhu et al., 2022). This was probably because high temperature stress enhanced the cell membrane permeability of roots and raised their levels of reactive oxygen species, thus, inhibiting the development of root hairs (Kim et al., 2021). Further research is merited to identify the specific cause.

## Conclusion

This study used a RhizoPot platform to investigate the dynamic traits of the root phenotypes of cotton under high temperature. When cotton was subjected to high temperature stress, its roots responded first. High temperature stress reduced the root length, root surface area, root volume, average root diameter, root growth rate, and root length density of cotton. The specific root length, root surface area, and root volume increased significantly, while the proportion of fine roots presented an opposite trend. After the removal of high temperature, the root length, average root diameter, and root growth rate all rejuvenated somewhat. The root hairs were more sensitive to high temperature stress, which shortened their lifespan and reduced the density and average length of root hairs. Cotton mainly adapts to high temperature stress by increasing the proportion of very fine roots, specific root length, and specific root surface area, which shortens the lifespan of root hairs.

## Data availability statement

The raw data supporting the conclusions of this article will be made available by the authors, without undue reservation.

## Author contributions

LL, YZ and CL initiated and designed the study. CF, MH, PS, HS and KZ performed the experiments and collected the data. CF, MH, and LL wrote the code and tested the methods. CF, ZB, GW, and HS analyzed the data and wrote the manuscript. All authors contributed to the article and approved the submitted version.

## Funding

This study was supported by grants from the National Natural Science Foundation of China (No. 32172120, 31871569), Natural Science Foundation of Hebei Province (C2022204158).

## Conflict of interest

The authors declare that the research was conducted in the absence of any commercial or financial relationships that could be construed as a potential conflict of interest.

## Publisher’s note

All claims expressed in this article are solely those of the authors and do not necessarily represent those of their affiliated organizations, or those of the publisher, the editors and the reviewers. Any product that may be evaluated in this article, or claim that may be made by its manufacturer, is not guaranteed or endorsed by the publisher.

## References

[B1] Alsajri F. A. Singh B. Wijewardana C. Irby J. T. Gao W. Reddy K. R. (2019). Evaluating soybean cultivars for low-and high-temperature tolerance during the seedling growth stage. Agronomy 9, 13. doi: 10.3390/agronomy9010013

[B2] Bates T. R. Lynch J. P. (2001). Root hairs confer a competitive advantage under low phosphorus availability. Plant Soil 236, 243–250. doi: 10.1023/A:1012791706800

[B3] Bengough A. G. McKenzie B. M. Hallett P. D. Valentine T. A. (2011). Root elongation, water stress, and mechanical impedance: a review of limiting stresses and beneficial root tip traits. J. Exp. Bot. 62, 59–68. doi: 10.1093/jxb/erq350 21118824

[B4] Benlloch-Gonzalez M. Bochicchio R. Berger J. Bramley H. Palta J. A. (2014). High temperature reduces the positive effect of elevated CO_2_ on wheat root system growth. Field Crops Res. 165, 71–79. doi: 10.1016/j.fcr.2014.04.008

[B5] Calleja-Cabrera J. Boter M. Oñate-Sánchez L. Pernas M. (2020). Root growth adaptation to climate change in crops. Front. Plant Sci. 11. doi: 10.3389/fpls.2020.00544 PMC7227386 32457782

[B6] Cheng X. Huang M. Shao M. Warrington D. N. (2009). A comparison of fine root distribution and water consumption of mature *Caragana korshinkii* kom grown in two soils in a semiarid region, China. Plant Soil 315, 149–161. doi: 10.1007/s11104-008-9739-5

[B7] Comas L. H. Eissenstat D. M. Lakso A. N. (2000). Assessing root death and root system dynamics in a study of grape canopy pruning: research assessing root mortality and decomposition. New Phytol. 147, 171–178. doi: 10.1046/j.1469-8137.2000.00679.x

[B8] Eissenstat D. M. Wells C. E. Yanai R. D. Whitbeck J. L. (2000). Building roots in a changing environment: implications for root longevity. New Phytol. 147, 33–42. doi: 10.1046/j.1469-8137.2000.00686.x

[B9] Fahad S. Bajwa A. A. Nazir U. Anjum S. A. Farooq A. Zohaib A.. (2017). Crop production under drought and heat stress: plant responses and management options. Front. Plant Sci. 8 1147. doi: 10.3389/fpls.2017.01147 28706531 PMC5489704

[B10] Fonseca de Lima C. F. Kleine-Vehn J. De Smet I. Feraru E. (2021). Getting to the root of belowground high temperature responses in plants. J. Exp. Bot. 72, 7404–7413. doi: 10.1093/jxb/erab202 33970267

[B11] Gahoonia T. S. Nielsen N. E. (1991). A method to study zhizosphere processes in thin soil layers of different proximity to roots. Plant Soil 135, 143–146. doi: 10.1007/BF00014787

[B12] Gao M. Xu B. Wang Y. Zhou Z. Hu W. (2021). Quantifying individual and interactive effects of elevated temperature and drought stress on cotton yield and fibre quality. J. Agron. Crop Sci. 207, 422–436. doi: 10.1111/jac.12462

[B13] Gavelienė V. Jurkonienė S. Jankovska-Bortkevič E. Švegždienė D. (2022). Effects of elevated temperature on root system development of two lupine species. Plants 11, 192. doi: 10.3390/plants11020192 35050080 PMC8777784

[B14] Giri A. Heckathorn S. Mishra S. Krause C. (2017). Heat stress decreases levels of nutrient-uptake and -assimilation proteins in tomato roots. Plants 6, 6. doi: 10.3390/plants6010006 PMC5371765 28106834

[B15] Hendrick R. L. Pregitzer K. S. (1992). The demography of fine roots in a northern hardwood forest. Ecology 73, 1094–1104. doi: 10.2307/1940183

[B16] Hoegh-Guldberg O. Jacob D. Taylor M. Guillén Bolaños T. Bindi M. Brown S.. (2019). The human imperative of stabilizing global climate change at 1.5°C. Science 365, eaaw6974. doi: 10.1126/science.aaw6974 31604209

[B17] Hund A. Fracheboud Y. Soldati A. Stamp P. (2008). Cold tolerance of maize seedlings as determined by root morphology and photosynthetic traits. Eur. J. Agron. 28, 178–185. doi: 10.1016/j.eja.2007.07.003

[B18] Joshi M. Fogelman E. Belausov E. Ginzberg I. (2016). Potato root system development and factors that determine its architecture. J. Plant Physiol. 205, 113–123. doi: 10.1016/j.jplph.2016.08.014 27669493

[B19] Jumrani K. Bhatia V. S. Pandey G. P. (2017). Impact of elevated temperatures on specific leaf weight, stomatal density, photosynthesis and chlorophyll fluorescence in soybean. Photosynth. Res. 131, 333–350. doi: 10.1007/s11120-016-0326-y 28025729

[B20] Kaplan E. L. Meier P. (1958). Nonparametric estimation from incomplete observations. J. Am. Stat. Assoc. 53, 457–481. doi: 10.1080/01621459.1958.10501452

[B21] Karlova R. Boer D. Hayes S. Testerink C. (2021). Root plasticity under abiotic stress. Plant Physiol. 187, 1057–1070. doi: 10.1093/plphys/kiab392 34734279 PMC8566202

[B22] Kim J. H. Castroverde C. D. M. Huang S. Li C. Hilleary R. Seroka A.. (2022). Increasing the resilience of plant immunity to a warming climate. Nature 607, 339–344. doi: 10.1038/s41586-022-04902-y 35768511 PMC9279160

[B23] Kim D. Yang J. Gu F. Park S. Combs J. Adams A.. (2021). A temperature-sensitive *FERONIA* mutant allele that alters root hair growth. Plant Physiol. 185, 405–423. doi: 10.1093/plphys/kiaa051 33721904 PMC8133571

[B24] Koevoets I. T. Venema J. H. Elzenga J. Theo. M. Testerink C. (2016). Roots withstanding their environment: exploiting root system architecture responses to abiotic stress to improve crop tolerance. Front. Plant Sci. 07. doi: 10.3389/fpls.2016.01335 PMC5005332 27630659

[B25] Kurogane T. Tamaoki D. Yano S. Tanigaki F. Shimazu T. Kasahara H.. (2021). Visualization of arabidopsis root system architecture in 3D by refraction-contrast X-ray micro-computed tomography. Microscopy 70, 536–544. doi: 10.1093/jmicro/dfab027 34264299

[B26] Li A. Zhu L. Xu W. Liu L. Teng G. (2022). Recent advances in methods for *in situ* root phenotyping. PeerJ 10, e13638. doi: 10.7717/peerj.13638 35795176 PMC9252182

[B27] Li X. Shi W. Broughton K. Smith R. Sharwood R. Payton P. (2020). Impacts of growth temperature, water deficit and heatwaves on carbon assimilation and growth of cotton plants (*Gossypium hirsutum* L.). Environ. Exp. Bot 179, 104204. doi: 10.1016/j.envexpbot.2020.104204

[B28] López-Bucio J. Cruz-Ramıírez A. Herrera-Estrella L. (2003). The role of nutrient availability in regulating root architecture. Curr. Opin. Plant Biol. 6, 280–287. doi: 10.1016/S1369-5266(03)00035-9 12753979

[B29] Luo Q. (2011). Temperature thresholds and crop production: a review. Climatic Change 109, 583–598. doi: 10.1007/s10584-011-0028-6

[B30] Luo H. Xu H. Chu C. He F. Fang S. (2020). High temperature can change root system architecture and intensify root interactions of plant seedlings. Front. Plant Sci. 11. doi: 10.3389/fpls.2020.00160 PMC7054236 32161613

[B31] Lynch J. P. (2011). Root phenes for enhanced soil exploration and phosphorus acquisition: tools for future crops. Plant Physiol. 156, 1041–1049. doi: 10.1104/pp.111.175414 21610180 PMC3135935

[B32] Mahmud K. P. Smith J. P. Rogiers S. Y. Nielsen S. Guisard Y. Holzapfel B. P. (2019). Diurnal dynamics of fine root growth in grapevines. Sci. Hortic-Amsterdam 250, 138–147. doi: 10.1016/j.scienta.2019.02.035

[B33] Mai W. Xue X. Feng G. Yang R. Tian C. (2018). Can optimization of phosphorus input lead to high productivity and high phosphorus use efficiency of cotton through maximization of root/mycorrhizal efficiency in phosphorus acquisition? Field Crops Res. 216, 100–108. doi: 10.1016/j.fcr.2017.11.017

[B34] Majeed S. Rana I. A. Mubarik M. S. Atif R. M. Yang S.-H. Chung G.. (2021). Heat stress in cotton: a review on predicted and unpredicted growth-yield anomalies and mitigating breeding strategies. Agronomy 11 , 1825. doi: 10.3390/agronomy11091825

[B35] Martins S. Montiel-Jorda A. Cayrel A. Huguet S. Roux C. P.-L. Ljung K.. (2017). Brassinosteroid signaling-dependent root responses to prolonged elevated ambient temperature. Nat. Commun. 8, 309. doi: 10.1038/s41467-017-00355-4 28827608 PMC5567177

[B36] McMichael B. L. Burke J. J. (1994). Metabolic activity of cotton roots in response to temperature. Environ. Exp. Bot. 34, 201–206. doi: 10.1016/0098-8472(94)90039-6

[B37] McMichael B. L. Quisenberry J. E. (1993). The impact of the soil environment on the growth of root systems. Environ. Exp. Bot. 33, 53–61. doi: 10.1016/0098-8472(93)90055-K

[B38] McMichael B. L. Upchurch D. R. Burke J. J. (1996). Soil temperature derived prediction of root density in cotton. Environ. Exp. Bot. 36, 303–312. doi: 10.1016/0098-8472(96)01015-5

[B39] Mohammad P. Shiraishi M. (2000). SEM observations on the root hairs of trifoliate orange grafted with satsuma mandarin under low soil moisture and high summer temperature condition. Pak. J. Biol. Sci. 3, 219–222. doi: 10.3923/pjbs.2000.219.222

[B40] Moore C. E. Meacham-Hensold K. Lemonnier P. Slattery R. A. Benjamin C. Bernacchi C. J.. (2021). The effect of increasing temperature on crop photosynthesis: from enzymes to ecosystems. J. Exp. Bot. 72, 2822–2844. doi: 10.1093/jxb/erab090 33619527 PMC8023210

[B41] Nagel K. A. Kastenholz B. Jahnke S. van Dusschoten D. Aach T. Mühlich M.. (2009). Temperature responses of roots: impact on growth, root system architecture and implications for phenotyping. Funct. Plant Biol. 36, 947. doi: 10.1071/FP09184 32688706

[B42] Nestler J. Wissuwa M. (2016). Superior root hair formation confers root efficiency in some, but not all, rice genotypes upon p deficiency. Front. Plant Sci. 7. doi: 10.3389/fpls.2016.01935 PMC5174101 28066487

[B43] Oliveira R. S. Bezerra L. Davidson E. A. Pinto F. Klink C. A. Nepstad D. C.. (2005). Deep root function in soil water dynamics in cerrado savannas of central Brazil. Funct. Ecology. 19, 574–581. doi: 10.1111/j.1365-2435.2005.01003.x

[B44] Otsuka K. Sugiyama M. (2012). Tissue organization of fasciated lateral roots of arabidopsis mutants suggestive of the robust nature of outer layer patterning. J. Plant Res. 125, 547–554. doi: 10.1007/s10265-011-0471-5 22258701

[B45] Pardales J. R. Kono Y. Yamauchi A. (1992). Epidermal cell elongation in sorghum seminal roots exposed to high root-zone temperature. Plant Sci. 81, 143–146. doi: 10.1016/0168-9452(92)90035-K

[B46] Parker L. E. McElrone A. J. Ostoja S. M. Forrestel E. J. (2020). Extreme heat effects on perennial crops and strategies for sustaining future production. Plant Sci. 295, 110397. doi: 10.1016/j.plantsci.2019.110397 32534613

[B47] Peterson R. L. Farquhar M. L. (1996). Root hairs: specialized tubular cells extending root surfaces. Bot. Rev. 62, 1–40. doi: 10.1007/BF02868919

[B48] Raven J. A. Edwards D. (2001). Roots: evolutionary origins and biogeochemical significance. J. Exp. Bot. 52, 381–401. doi: 10.1093/jxb/52.suppl_1.381 11326045

[B49] Reddy K. R. Brand D. Wijewardana C. Gao W. (2017). The effects of high temperature stress on some agronomic characters in cotton. Agron. J. 109, 1379–1387. doi: 10.2134/agronj2016.07.0439

[B50] Ribeiro P. R. Fernandez L. G. de Castro R. D. Ligterink W. Hilhorst H. W. (2014). Physiological and biochemical responses of *Ricinus communis* seedlings to different temperatures: a metabolomics approach. BMC Plant Biol. 14, 223. doi: 10.1186/s12870-014-0223-5 25109402 PMC4236761

[B51] Shen C. Liu L. Zhu L. Kang J. Wang N. Shao L. (2020). High-throughput *in situ* root image segmentation based on the improved deepLabv3+ method. Front. Plant Sci. 11. doi: 10.3389/fpls.2020.576791 PMC7604297 33193519

[B52] Snider J. L. Thangthong N. Rossi C. Pilon C. (2022). Root system growth and anatomy of cotton seedlings under suboptimal temperature. J. Agron. Crop Sci. 208, 372–383. doi: 10.1111/jac.12591

[B53] Tahir I. S. A. Nakata N. Yamaguchi T. Nakano J. Ali A. M. (2008). Influence of high shoot and root-zone temperatures on growth of three wheat genotypes during early vegetative stages. J. Agron. Crop Sci. 194, 141–151. doi: 10.1111/j.1439-037X.2008.00298.x

[B54] Taranet P. Kirchhof G. Fujinuma R. Menzies N. (2018). Root zone temperature alters storage root formation and growth of sweetpotato. J. Agron. Crop Sci. 204, 313–324. doi: 10.1111/jac.12262

[B55] Tiwari M. Kumar R. Min D. Jagadish S. V. K. (2022). Genetic and molecular mechanisms underlying root architecture and function under heat stress–a hidden story. Plant Cell Environ. 45, 771–788. doi: 10.1111/pce.14266 35043409

[B56] Virk G. Snider J. L. Chee P. Jespersen D. Pilon C. Rains G.. (2021). Extreme temperatures affect seedling growth and photosynthetic performance of advanced cotton genotypes. Ind. Crop Prod. 172, 114025. doi: 10.1016/j.indcrop.2021.114025

[B57] Wang L. Li X. Mang M. Ludewig U. Shen J. (2021). Heterogeneous nutrient supply promotes maize growth and phosphorus acquisition: additive and compensatory effects of lateral roots and root hairs. Annal. Bot. 128, 431–440. doi: 10.1093/aob/mcab097 PMC8414595 34309655

[B58] Wang W. Vinocur B. Altman A. (2003). Plant responses to drought, salinity and extreme temperatures: towards genetic engineering for stress tolerance. Planta 218, 1–14. doi: 10.1007/s00425-003-1105-5 14513379

[B59] Wei F. Fanella B. Guo L. Wang X. (2016). Membrane glycerolipidome of soybean root hairs and its response to nitrogen and phosphate availability. Sci. Rep. 6, 1–11. doi: 10.1038/srep36172 27812010 PMC5095881

[B60] Weng J. Rehman A. Li P. Chang L. Zhang Y. Niu Q. (2022). Physiological and transcriptomic analysis reveals the responses and difference to high temperature and humidity stress in two melon genotypes. Int. J. Mol. Sci. 23, 734. doi: 10.3390/ijms23020734 35054918 PMC8776189

[B61] Xiao S. Liu L. Zhang Y. Sun H. Zhang K. Bai Z.. (2020). Fine root and root hair morphology of cotton under drought stress revealed with RhizoPot. J. Agro. Crop Sci. 206, 679–693. doi: 10.1111/jac.12429

[B62] Xu B. Zhou Z. Guo L. Xu W. Zhao W. Chen B.. (2017). Susceptible time window and endurable duration of cotton fiber development to high temperature stress. J. Integr. Agr. 16, 1936–1945. doi: 10.1016/S2095-3119(16)61566-6

[B63] Zahid K. R. Ali F. Shah F. Younas M. Shah T. Shahwar D.. (2016). Response and tolerance mechanism of cotton *Gossypium hirsutum* l. @ to elevated temperature stress: a review. Front. Plant Sci. 7. doi: 10.3389/fpls.2016.00937 PMC4927942 27446165

[B64] Zeleznik J. D. Dickmann D. I. (2004). Effects of high temperatures on fine roots of mature red pine (*Pinus resinosa*) trees. For. Ecol. Manage. 199, 395–409. doi: 10.1016/j.foreco.2004.05.050

[B65] Zhang C. Stratópoulos L. M. F. Xu C. Pretzsch H. Rötzer T. (2020). Development of fine root biomass of two contrasting urban tree cultivars in response to drought stress. Forests 11, 108. doi: 10.3390/f11010108

[B66] Zhang R. Zhou L. Li Y. Ma H. Li Y. Ma Y.. (2022). Rapid identification of pollen- and anther-specific genes in response to high-temperature stress based on transcriptome profiling analysis in cotton. Int. J. Mol. Sci. 23, 3378. doi: 10.3390/ijms23063378 35328797 PMC8954629

[B67] Zhang Z. Zhu L. Li D. Wang N. Sun H. Zhang Y.. (2021). *In situ* root phenotypes of cotton seedlings under phosphorus stress revealed through RhizoPot. Front. Plant Sci. 12. doi: 10.3389/fpls.2021.716691 PMC8435733 34527012

[B68] Zhen B. Li H. Niu Q. Qiu H. Tian G. Lu H.. (2020). Effects of combined high temperature and waterlogging stress at booting stage on root anatomy of rice (*Oryza sativa* l.). Water 12, 2524. doi: 10.3390/w12092524

[B69] Zhu L. Liu L. Sun H. Zhang Y. Liu X. Wang N.. (2022). The responses of lateral roots and root hairs to nitrogen stress in cotton based on daily root measurements. J. Agron. Crop Sci. 208, 89–105. doi: 10.1111/jac.12525

